# Effects of Difficulty in Handling Emotions and Social Interactions on Nomophobia: Examining the Mediating Role of Feelings of Loneliness

**DOI:** 10.1007/s11469-022-00888-w

**Published:** 2022-07-28

**Authors:** Giusy D. Valenti, Rossella Bottaro, Palmira Faraci

**Affiliations:** 1 Department of Psychology, Educational Sciences and Human Movement, 90128 Palermo, Italy; 2 Faculty of Human and Social Sciences, University of Enna “Kore”, Enna, Italy

**Keywords:** Emotion regulation, Loneliness, Mediation analysis, Nomophobia, Social interaction anxiety

## Abstract

This study was addressed to assess nomophobia in an Italian sample (*N* = 456, 53.1% men, *M*_*age*_ = 31.8, *SD* = 11.1), also providing a deeper knowledge about how it is distributed across demographics, as well as identifying its best predictors. The main goal was to investigate the direct and indirect effects of difficulty in emotion regulation and social interaction anxiety on nomophobia through loneliness. Our findings indicated that loneliness explained the effect of the expressive suppression strategy (fully) and social interaction anxiety (partially) on nomophobia, whereas it was not a significant mediator when the cognitive reappraisal strategy was taken into account. Our study suggests that loneliness during the pandemic plays a crucial role in explaining the associations between the investigated predictors and the outcome variable, offering a deeper understanding of the underlying mechanisms of this emerging construct. Theoretical and practical implications are discussed, and shortcomings and suggestions for future works are presented.

Technological development has rapidly proceeded in these last years, leading to a constant change in people’s lifestyles. With the introduction of mobile technologies, new forms of behaviors, as well as new forms of communication and interaction, have begun to develop (Gurbuz & Ozkan, 2020). Providing several advantages (such as online shopping, social networks, GPS navigation, games, videos), smartphones use has exponentially increased, representing an indispensable component for all individuals’ lives, both for youngest and for oldest (Bush et al., 2021; Gonçalves et al., 2020).

Despite these advantages, the frequent and massive use of smartphones may have short-term and long-term adverse effects on psychological wellbeing, altering levels of attention and concentration, or provoking psychological disorders such as Internet, smartphones, gaming, or social media addictions, as well as depression and anxiety (Bragazzi & Del Puente, 2014; Deursen et al., 2015; Elhai et al., 2017; Mendoza et al., 2018). Particularly, the use of smartphones is strictly related to the use of social network sites, whose problematic use negatively impacts several mental health outcomes, in terms of addictive behaviors and detrimental psychological states due to the negative upward social comparisons (Gonzáles-Nuevo et al., 2021; Keles et al., 2020), together with consequences on sleep quality and ADHD (Hussain & Griffiths, 2019).

A further negative consequence of the problematic use of smartphone is nomophobia—acronym for “no mobile phone phobia”—which is referred to a sense of discomfort, anxiety, and nervousness of being without a mobile phone, of becoming unable to communicate through a mobile device, of being unable to get information through Internet, and giving up the convenience that smartphones provide (Yldirim & Correia, 2015). People higher in nomophobia become anxious when they forget their own smartphone, when the battery is discharged, or when there is not Internet coverage (Gezgin et al., 2018a).

Recent systematic reviews (León-Mejia et al., 2021; Rodriguéz-Garcia et al., 2020) indicated that current research on this topic is in an exploratory phase, with a significant number of descriptive, cross-sectional, and nonexperimental studies examining the prevalence of nomophobia among individuals, and that this emerging construct led to negative consequences on personality, self-esteem, anxiety, stress, academic performance, and other physical and psychological health outcomes. According to a recent meta-analysis (Humood et al., 2021), the prevalence of moderate to severe nomophobia exceeds 70%, and approximately 20% of general population shows severe nomophobic symptoms. From this perspective, it is noticeable that nomophobia is a public health concern and that its understanding and examination deserve attention.

Evidence from some previous studies indicated that nomophobia is not equally distributed across sociodemographic variables. In fact, although it seems to affect individuals regardless of their own gender, age, or educational level (Argumosa-Villar et al., 2017; Yldirim et al., 2016), some studies showed that women, youngers, and less educated people tend to report higher nomophobic symptoms than men, olders, and people with a higher education level (Arpaci, 2020; Gezgin et al., 2018a; Gonzáles-Cabrera et al., 2017; Gurbuz & Ozkan, 2020; Ma & Liu, 2018).

## Nomophobia and Its Associations with Emotion Regulation, Social Interaction Anxiety, and Loneliness

Some authors have linked nomophobia to the difficulties in emotion regulation (Delavarpour et al., 2019; Ercenzig et al., 2020; Gonçalves et al., 2020), highlighting that people with a poor ability in remodulating a situation, changing its meaning and emotional impact (cognitive reappraisal), and/or in inhibiting or reducing emotion-expressive behaviors (expressive suppression) tend to use their smartphones as a source of assurance and relief and a mean to escape from the emotive problems experienced in the real world, also reporting a maladaptive smartphone usage (Horwood & Anglim, 2021); consequently, when out of their mobile devices, these people are more prone to express irritability, nervousness, or anxiety, thus showing nomophobic symptoms.

Following, other scholars have associated nomophobia with social interaction anxiety (Anshari et al., 2019; Kaur et al., 2021). Indeed, highly socially anxious individuals prefer virtual relationships to face-to-face interactions in order to avoid feelings of uncomfortableness and unease (Shalom et al., 2015). From this point of view, the unavailability of accessing to smartphones or the thought of being out of a mobile device can be seen as a source of anxiety by people with high levels of social interaction anxiety, because the use of smartphones is a sort of coping strategy which helps them to ease social discomfort (Brown & MedCalf-Bell, 2022).

Further, Ercenzig et al. (2020), along with Brown and MedCalf-Bell (2022), evidenced that both poor emotion management skills and social interaction anxiety indirectly affected nomophobia through the intolerance of uncertainty. This means that both individuals with deficits in regulating their own emotions and those with scarce abilities in handling in-person social interactions tend to show higher levels of intolerance of uncertainty, and, in both situations, the attachment to and the dependence on smartphones increase as a way to alleviate unpleasant feelings.

Nomophobia has also been related to feelings of loneliness (Arpaci, 2020; Gezgin et al., 2018b; Nguyen et al., 2022). Several studies had previously identified loneliness as a potential risk factor for the development of problematic smartphone and Internet usage or smartphone and Internet addiction, because individuals who self-identify as lonely tend to use their smartphones more frequently, thanks to their benefits of reducing the discomfort of feeling lonely (Bian & Leung, 2014; Billieux, 2012).

## Nomophobia During the Pandemic

Various studies have been conducted during the global health crisis on nomophobia: For instance, Bhatnagar et al. (2021) assessed nomophobia prior to and during the COVID-19 lockdown, reporting an increase of smartphone overuse during the pandemic restrictions, with a significant proportion of participants with moderate to severe nomophobic symptoms, whereas Sui et al. (2022) indicated that nomophobic symptoms tend to be stable when compared with pre-pandemic data; also, Zwilling (2022) showed that nomophobic levels, along with their repercussions on problematic smartphone use, increased from the beginning of the first COVID-19 wave (T1) to the end of the COVID-19 lockdown (T2), highlighting how the addictive outcomes endure longer, even when the pandemic was less widespread; further, Nguyen et al. (2022) investigated the effects of nomophobia on stress, also testing the indirect path through loneliness, indicating that the perception of feeling lonely may increase stress in individuals who show nomophobic symptoms.

## Research Goals and Hypotheses

The primary aim of the current study was to assess nomophobia in an Italian sample during the COVID-19 pandemic, also providing a general knowledge about its distribution across demographics, as well as identifying its best predictors.

**Fig. 1:**
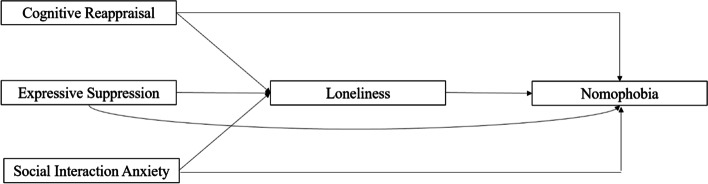
The proposed mediation model

We expected to find a direct effect of emotion regulation (in its two components) and social interaction anxiety on nomophobia, as reported in previous research (Anshari et al., 2019; Delavarpour et al., 2019; Ercenzig et al., 2020; Gonçalves et al., 2020; Kaur et al., 2021). Further, a mediation model in which loneliness was introduced as a mediator in the relationship between social interaction anxiety, emotion regulation, and nomophobia was proposed (Figure 1). In other words, it was hypothesized that loneliness could provide an explanation about “how” and “why” the effect of social interaction anxiety and emotion regulation on nomophobia occurs. Because such a mediation model was not previously examined, our study could improve the understanding of the phenomenon, providing a contribution to the existing and developing literature. The path from emotion regulation to loneliness (Eres et al., 2021; Kearns & Creaven 2017), as well as from social interaction anxiety to loneliness (Cacioppo et al., 2015; Eres et al., 2021; Fung et al., 2017; Lim et al., 2016), is well documented in literature, thus providing support to the choice of the hypothesized sequential relationships.

Identifying the major risk factors for the development of nomophobic symptoms—especially in a period characterized by a growth in the problematic and addictive usage of smartphones—becomes a relevant topic both for theoretical and practical goals.

## Method

### Participants and Procedure

A total of 456 participants (53.1% men), aged from 18 to 67 (*M =* 31.8; *SD* = 11.1), was recruited through Amazon Mechanical Turk (MTurk). Among them, 44.3% had a basic education level (from 5 to 13 years of education), whereas 55.7% had a higher education level (from 13 to 21 years of education). As requirements for participation, we set (1) a HIT approval rate greater than or equal to 90, (2) Italy as location, and (3) age 18 and over. No additional criteria were specified. Upon completion of the survey, each respondent received .40 €. We closed the HIT after 1 week. A set of standardized questionnaires addressed to measure the study variables was used; instruments were administered in a random fashion to avoid the effects of order and sequence. Participants were assured that data would be collected and used only for research purposes and analyzed collectively. Informed consent was given by all participants prior to answering the survey. A preliminary inspection of data showed that the percentage of missing data was less than .5% in all of the investigated variables. Thus, missing data were handled by applying the mean imputation technique, which can be considered an adequate procedure when the percentage of missing data is low and when data are missing completely at random (MCAR) (Cook, 2021; Parent, 2012).

### Instruments

#### Nomophobia Questionnaire

The Nomophobia Questionnaire (NMP-Q; Yldirim & Correia, 2015) was used to assess nomophobia, that is the sense of discomfort, anxiety, and nervousness of being without a mobile phone, of becoming unable to communicate through a mobile device, of being unable to get information through Internet, and of giving up the convenience that smartphones provide (Yldirim & Correia, 2015). In the current study the Italian version (Adawi et al., 2018) was applied. It is composed of 20 items rated on a 7-point Likert scale (from 1 “strongly disagree” to 7 “strongly agree”). It consists of four subscales: (1) not being able to communicate, (2) losing connectedness, (3) not being able to access information, and (4) giving up convenience. It also allows to provide a total score of nomophobia, by summing up all 20 items. In this study, the global level of nomophobia was taken into account. Following the procedure proposed by Yldirim and Correia (2015), nomophobia scores are interpreted as follows: A score of 20 reflects absence of nomophobia; NMP-Q scores between 20 and 59 indicate mild levels of nomophobia; NMP-Q scores between 60 and 99 are indicative of moderate levels of nomophobia; NMP-Q scores higher than 100 correspond to severe nomophobia. The internal reliability for the current study sample was excellent (*α* = .95).

#### UCLA Loneliness Scale Version 3

The UCLA Loneliness Scale Version 3 (UCLA LS3; Russell; 1996) was used to assess loneliness, meant as an unpleasant feeling due to the subjective perception of social isolation (Hawkley & Cacioppo, 2010). In the current study, the Italian version (Boffo et al., 2012) was applied. The UCLA LS3 is a unidimensional scale, composed of 20 items on a 4-point Likert (from 1 “never” to 4 “always”). Higher scores are indicative of greater levels of feelings of loneliness. The internal reliability for the current study sample was excellent (*α* = .90).

#### Emotion Regulation Questionnaire

To evaluate the ability of managing emotion, the Italian version (Balzarotti et al., 2010) of the Emotion Regulation Questionnaire (ERQ; Gross & John, 2003) was used. It is composed of 10 items distributed into two subscales, reflecting two different strategies of emotion regulations: cognitive reappraisal (items 1, 3, 5, 7, 8, 10), which describes difficulties in remodulating and changing an unpleasant situation, and expressive suppression (items 2, 4, 6, 9), which indicates problems in reducing ongoing expressive behaviors. Good to excellent were the Cronbach’s alpha values in the present study (*α* = .90 for cognitive reappraisal; *α* = .78 for expressive suppression). Higher scores suggest greater emotion dysregulation.

#### Social Interaction Anxiety Scale

The Social Interaction Anxiety Scale (SIAS; Mattick & Clarke, 1998) was used to assess social interaction anxiety, meant as “the fear and avoidance of meeting, interacting, and expressing oneself with others” (Kashdan, 2004, p. 721). It is composed of 19 with a 5-point Likert-type scale (from 0 “not at all” to 4 “extremely”). In this study, the Italian version of the scale was applied (Sica et al., 2007). Higher scores indicate greater levels of social interaction anxiety. The level of internal consistency for the current study sample was excellent (*α* = .94).

### Data Analyses

Data analyses were performed using jamovi 1.6.23, with the application of jAMM (jamovi advanced mediation model) statistical package to test the mediation analysis (Gallucci, 2019). For the preliminary analyses, descriptive statistics were computed, univariate normal distribution was verified, and correlation analyses among the study variables were performed to exclude the presence of multicollinearity. Arguably, with correlation coefficients below .80, it is possible to proceed with the analyses assuming relative independence among the variables. To verify whether NMP-Q scores differed across demographics, independent samples *t test* were conducted. The median split was used to dichotomize age into two categories (young adults, equal to or less than 30 years; adults, more than 30 years). A common and useful practice in psychological research is to turn continuous to categorical variables in order to simplify the analyses and the interpretation of the results (DeCoster et al., 2011). The median split was chosen because this procedure allows to turn a continuous variable into a dichotomous variable, in which each group (above and below the median value) has the same sample size.

Cohen’s *d* was also calculated to provide the effect size. Prior to testing the mediating model, the multivariate normality distribution of data was checked through the Mahalanobis Distance computation. Since the Mardia’s coefficient (38.21) exceeds the critical value associated with five-degrees-of-freedom (35), the assumption of multivariate normality was not met. Therefore, the bootstrapping (percentiles), a non-parametric resampling procedure, was applied, which is recognized as a robust and accurate method for mediation analysis (Carríon et al., 2017) and the best suited technique to be performed when the multivariate normality of data is violated. In order to verify whether our sample size was adequate for conducting our analyses, Fritz and MacKinnon’s (2007) study was taken into account, according to which a sample size of at least 78 is needed to achieve .80 power for a medium-medium (*α* = .39 and *β* = .39) condition.

## Results

### Preliminary Analyses

Descriptive statistics and correlations between the study variables are shown in Table 1. Both skewness and kurtosis indices did not exceed the value of |1|, indicating that all the selected variables showed a univariate normal distribution. In addition, scores on NMP-Q obtained by our sample were 75.5 (*SD=* 25.8), indicating a moderate level of nomophobia according to the cutoff suggested by Yldirim and Correia (2015). Among the inspected relationships between the study variables, only four of them were moderate (.368 < *r* < .632, *p* < .001).

**Table 1:** Descriptive statistics and correlation between nomophobia, emotion regulation, loneliness, and social interaction anxiety

Measure	*M*	*SD*	*S*	*K*	1	2	3	4	5
1. NMQP-Q	75.5	25.8	.010	−.514	-				
2. EQR cognitive reappraisal	29.3	6.96	−.509	.359	.053	-			
3. EQR expressive suppression	16.9	5.00	−.178	.392	.181***	.142**	-		
4. SIAS	33.2	16.4	.141	−.734	.429***	.129**	.368***	-	
5. UCLA LS-3	48.1	10.3	.128	−.204	.191***	.091*	.390***	.632***	-

*NMP-Q* Nomophobia Questionnaire, *ERQ* Emotion Regulation Questionnaire, *UCLA-LS3* UCLA Loneliness Scale Version 3, *IAS* Social interaction anxiety

^*^p <.05; **p < .01; *p <.* 001

### Independent Sample *t-*test

By performing the independent samples *t-*test, a statistical mean difference in NMP-Q scores between people with a basic education level and those with a higher education level was found, with the former reporting lower level of nomophobic symptoms than the latter (basic education: *M* = 70.75, *SD* = 26.22; higher education: *M* = 79.19, *SD* = 24.85; *t*_(454)_ = 1.233, *p* < .001, *d* = .331). No statistical differences were estimated between men and women and between the two age groups (see Table 2 for more details).

**Table 2:** Differences in NMP-Q across demographic variables

Grouping variable	*N*	*M*	*SD*	*t*	df	*p*	*d*
Gender							
Men	242	74.37	25.8				
Women	214	76.68	25.8				
				−.953	.454	.341	.089
Age							
Young adults	241	76.86	23.9				
Adults	215	73.88	27.7				
				1.233	454	.218	.116
Education level							
Basic education	202	70.75	26.22				
Higher education	254	79.19	24.85				
				−.352	454	<. 001	.331

### Mediation Analysis

**Fig. 2:**
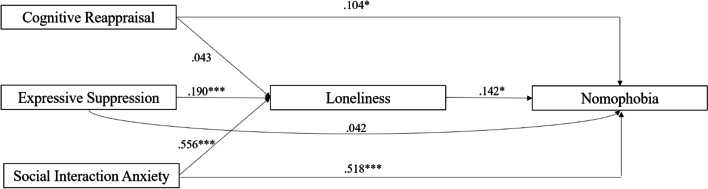
Direct and indirect effects of emotional regulation and social interaction anxiety on nomophobia. *p < .05; **p < .01; ***p < .001

The outcomes of the mediation analysis are reported in Table 3, presenting the standardized β and the confidence intervals (C.I.) 95%, which indicate the significance of the effect with a 5% of probability of error (C.I. that does not comprise 0 are significant). Findings from our analyses revealed that loneliness functioned as a full mediator in the relationship between ERQ Expressive Suppression and nomophobia (β = .026, *p* = .024, 95% CI [−0.015, −0.102]), as well as a partial mediator in the relationship between social interaction anxiety and nomophobia (β = .078, *p*=.012, 95% CI [−0.221, −0.027]). On the other hand, ERQ Cognitive Reappraisal directly affected nomophobia (β = .104, *p* =.016, 95% CI [0.070, 0.696]), but the path from ERQ Cognitive Reappraisal to nomophobia through loneliness was not significant (β = .006, *p* =.280, 95% CI [−0.018, 0.064]). The final model is shown in Fig. 2.

**Table 3:** Total, direct, and indirect associations of emotion regulation and social interaction anxiety through loneliness

**95% C.I. (a)**								
**Type**	**Effect**	**Estimate**	**SE**	**Lower**	**Upper**	**β**	**z**	***p***
Indirect	ERQ_Cognitive_Reappraisal ⇒ UCLA_LS_tot ⇒ NMPQ_tot	0.0229	0.0212	−0.0186	0.0644	0.00622	1.081	0.280
	ERQ_Expressive_Suppression ⇒ UCLA_LS_tot ⇒ NMPQ_tot	**0.1403**	**0.0623**	−**0.2624**	−**0.0183**	**0.02696**	**2.254**	**0.024**
	SIAS_tot ⇒ UCLA_LS_tot ⇒ NMPQ_tot	**0.1238**	**0.0494**	−**0.2206**	−**0.0270**	**0.07883**	**2.507**	**0.012**
Direct	ERQ_Cognitive_Reappraisal ⇒ NMPQ_tot	**0.3829**	**0.1595**	**0.0703**	**0.6955**	**0.10410**	**2.401**	**0.016**
	ERQ_Expressive_Suppression ⇒ NMPQ_tot	0.2196	0.2467	−0.2639	0.7032	0.04219	0.890	0.373
	SIAS_tot ⇒ NMPQ_tot	**0.8137**	**0.0873**	**0.6427**	**0.9847**	**0.51813**	**9.324**	**< .001**
Total	ERQ_Cognitive_Reappraisal ⇒ NMPQ_tot	**0.4058**	**0.1606**	**0.0911**	**0.7205**	**0.11032**	**2.527**	**0.011**
	ERQ_Expressive_Suppression ⇒ NMPQ_tot	0.0793	0.2425	−0.3960	0.5546	0.01523	0.327	0.744
	SIAS_tot ⇒ NMPQ_tot	**0.6899**	**0.0731**	**0.5467**	**0.8331**	**0.43930**	**9.440**	**< .001**

*NMP-Q* Nomophobia Questionnaire, *ERQ* Emotion Regulation Questionnaire, *UCLA-LS3* UCLA Loneliness Scale Version 3, *IAS* Social interaction anxiety

Significant associations are in bold

## Discussion

This study was primarily addressed to examine the prevalence of nomophobic symptoms in a sample of Italian individuals, also offering a general view about how nomophobic scores were distributed across demographic variables. On the whole, taking into account the cutoff values proposed by Yldirim & Correia (2015), our study sample reported a moderate level of nomophobia. Although not alarming, these results pointed out that this specific disorder is widespread, and without suitable measures aimed at preventing and/or reducing its diffusion, it may become higher and higher.

Regarding the influence of gender and age on nomophobia, our study contradicts findings from previous research (Arpaci, 2020; Galhardo et al., 2020; Gezgin et al., 2018a; Gonzáles-Cabrera et al., 2017; Ma & Liu, 2018) according to which women and younger individuals are more likely to show nomophobic symptoms. As a matter of fact, no statistical differences in NMP-Q scores were estimated as far as it concerns these two demographic variables. The absence of a significant effect of gender and age on nomophobia may be attributed to the specific period in which data were gathered which has changed indiscriminately the way in which both men and women and young adults and adults interact with their smartphones; thus, all people, regardless of their own gender and age, may share the same feelings—in terms of irritability, nervousness, anxiety, or discomfort—when the access to their smartphone is denied. However, some studies conducted prior to the COVID-19 diffusion (Argumosa-Villar et al., 2017; Yldirim and Correia, 2015, Yldirim et al., 2016) reported no statistical differences in nomophobic symptoms across gender or age, highlighting that nomophobia is equally prevalent among diverse gender and age groups. Similarly, results from a recent meta-analysis (Humood et al., 2021) indicated that neither gender nor age was a significant predictor of nomophobia, in line with our findings. So, these contrasting evidence seem to indicate that gender and age differences in nomophobia are still to be determined, suggesting the need for further investigation. From this perspective, León-Mejia et al. (2021), in their systematic review, emphasized that these incoherencies may be attributed to the methodological disparities among the examined studies, making difficult to establish any predictive role of gender and age on nomophobia.

On the other hand, more educated people reported a higher level of nomophobia, contrasting results from previous empirical research claiming that nomophobia decreases as education level increases (Gurbuz & Ozkan, 2020). Also these unexpected results could be read in the light of the critical period in which data were collected: More educated individuals—who are more likely workers—may use their mobile phones also for working purposes, such as emailing with colleagues and supervisors, getting information about work schedule or work shifts, or organizing meetings. So, it is plausible that more educated people—who presumably work in smart working—show a higher sense of discomfort and anxiety when the access to their smartphone is not available, because not being able to access to their mobile phone may cause a loss of relevant information. Future studies are needed to verify this assumption. However, also Darvishi et al. (2019) found greater nomophobic levels in people with a higher education level, thus providing further evidence that the role of sociodemographic variables in predicting nomophobia is somewhat doubtful and unclear.

Further, our study was aimed at testing the potential mediating role of loneliness in the relationship between emotion regulation (in its twofold components, cognitive reappraisal and expressive suppression), social interaction anxiety, and nomophobia. By examining the paths emerged from our proposed model, loneliness was found to partially mediate the association between social interaction anxiety and nomophobia. If the direct effect of social interaction anxiety on nomophobia indicates that individuals with higher grades of social interaction anxiety may be more vulnerable in developing nomophobic symptoms, the significant indirect effect confirmed our hypotheses, also supporting previous empirical evidence (Cacioppo et al., 2015; Fung et al., 2017; Lim et al., 2016) claiming that social anxiety plays a crucial role in loneliness onset and maintenance. Thus, it could be said that people higher in social interaction anxiety may show nomophobic symptoms also due to the sense of loneliness they perceive. Nevertheless, the magnitude of the examined associations was not very high, likely due to the specific period of data collection: Because of to the social isolation imposed by the governments to reduce the spread of the virus, social interactions and face-to-face contacts were extremely restricted, thus probably affecting the levels of social interaction anxiety. This could presumably mean that the reported levels of social interaction anxiety were lower than those individuals would have manifested in normal circumstances (i.e., in the absence of the pandemic restrictive measures). Indeed, during the pandemic, social networks were limited to partners, family members, or close friends, whereas contacts with larger and non-homogeneous groups were notably reduced (Long et al., 2022).

The associations between emotion regulation and nomophobia through loneliness gave rise to interesting results: As far as it concerns the expressive suppression strategy, loneliness functioned as a full mediator. This means that the difficulty in inhibiting or reducing emotion-expressive behaviors was not directly associated with nomophobic symptoms. As a viable explanation, because suppression, as reported by Balzarotti et al., (2010), is related to less experience, less social sharing, relationship closeness, and no repair efforts, people higher in this dimension are not likely interested in using their mobile phones for communicating with family and friends, for sharing their own feelings, or for finding comfort to their difficulty in handling their own emotions. From this perspective, it is reasonable that these individuals do not report anxiety, irritability, or nervousness when the access to their smartphone is not available. Thus, the effect of this emotion regulation strategy on nomophobia was totally explained by the occurrence of loneliness.

On the contrary, cognitive reappraisal directly affected nomophobic symptoms, while no indirect significant effects were estimated. It means that individuals who show difficulties in the try to think about a situation and in remodulating its meaning and its emotive impact tend to report nomophobic symptoms not because they feel lonely. Probably, people with a poor ability in cognitive reappraisal, who usually use rumination or self/other blaming in face of emotional difficulties, are more likely prone to seek online constant reassurance and relief, regardless of the occurrence of feelings of loneliness; thus, they may suffer when the access to their own smartphones is not available. These findings are in line with those reported in a previous study (Extremera et al., 2019) according to which rumination and self/other blame were successful predictors of problematic smartphone usage.

As practical implications, these findings indicate that the primary areas of interventions to prevent and reduce nomophobic symptoms should be addressed to lessen feelings of loneliness, helping people to seek in-person social support, enhancing the opportunities for social contacts, and also promoting educational programs or psychological interventions, such as mindfulness or cognitive-based interventions. Specifically, a recent systematic review is recommended (Williams et al., 2021) for a better comprehension of the most effective intervention programs to reduce loneliness during the COVID-19.

Adequate programs should be also tailored to help individuals to look on the bright side of the negative experiences, allowing a remodulation of the harmful emotional impacts. As an example, although the COVID-19 pandemic is undoubtedly an adverse experience, with negative emotional repercussions, focusing on some positive aspects, such as the availability to learn new abilities (during the home quarantine, a lot of people learnt new skills or fulfilled their hobbies), or spending more time with family, may help people to consider such a dramatic situation from a different perspective, decreasing the level of negative emotions.

The results from our study should be read in the light of some limitations. As a major concern, because the study variables were not assessed prior to the COVID-19 diffusion, it is not possible to affirm whether the levels of nomophobia, as well as the levels of the other variables, changed compared to the previous period. So, we cannot clearly state if the proposed model is adequate to explain the relationships among the examined variables only during the pandemic situation or if it may equally function also in other situations.

As a further limitation, we considered the global nomophobia score on NMP-Q, but not the score on each of its subscales. Although the application of a total nomophobia score is common across international literature (Gezgin et al., 2018a, 2018b; Kaviani et al., 2020), the examination of nomophobia subdimensions would have offered more detailed information about the associations among the investigated variables.

## Conclusion

Our study aimed at offering a deeper knowledge and understanding of nomophobia, providing evidence of its best predictors in a crucial and historical period, characterized by the diffusion of the COVID-19 pandemic. The novelty of the present work is its focus on loneliness as a potential mediator of the hypothesized relationships among the study variables. Our results suggest that loneliness partly or fully explains why the effects of difficulties in handling emotions and social interactions on nomophobia occur, also highlighting that is an unbearable feeling and a public concern in the era of COVID-19, as stated in a previous work (Padmanabhanunni & Pretorius, 2021). Our findings are in line with prior international literature (Killgore et al., 2020) which identified loneliness as the most significant mental health consequence of prolonged isolation and social distancing, with severe repercussion over individuals’ general health. Also, as indicated by a previous study (Zhen et al., 2021), feelings of loneliness experienced during the pandemic intensified problematic smartphone usage and mobile phone addiction. This may indicate that the moderate levels of nomophobic symptoms reported by our sample are mainly attributed to the perception of being lonely, a common and unpleasant feeling experienced during social isolation, as also reported in previous research (Faraci et al., 2022; Killgore et al., 2020; Padmanabhanunni & Pretorius, 2021).
